# Central Diabetes Insipidus Induced by Acute Myeloid Leukemia with DNMT3A Mutation

**DOI:** 10.1155/2022/2750146

**Published:** 2022-05-23

**Authors:** Priyanka Lakshmanan, Heena Asnani, David Knorr

**Affiliations:** ^1^Department of Medicine Mount Sinai Morningside, Mount Sinai West Icahn School of Medicine, New York, NY, USA; ^2^Pravara Institute of Medical Sciences, Ahmednagar, India; ^3^Memorial Sloan Kettering Cancer Center, New York, NY, USA

## Abstract

Central diabetes insipidus (CDI) is an uncommon complication of acute myeloid leukemia (AML). Patients present with polyuria either preceding or at the time of diagnosis of AML. We describe the case of a 36-year-old male who presented with a subacute onset of polyuria, polydipsia, nocturia, and fatigue. After an extensive workup, he was diagnosed with AML/CMML (chronic myelomonocytic leukemia) with a normal karyotype with DNMT3A, CBFB, and PTPN11 mutations. Further workup of the polyuria with a water deprivation test helped confirm the diagnosis of CDI along with MRI findings suggestive of hypophysitis. In this report, we analyze the clinical workup for AML and CDI and report the possibility of extramedullary leukemic infiltration associated with DNMT3A mutation, which has been reported as one of the mechanisms in the existing literature. We also discuss other theories hypothesized to cause CDI in AML patients with abnormal karyotypes. Our patient progressed to AML from CMML-2 after a cycle of decitabine, with confirmed gingival and presumed central nervous system (CNS) involvement. He is in minimal residual disease (MRD)-negative complete remission after induction with a CNS-active acute lymphoblastic leukemia-2 regimen. He also received double umbilical cord blood transplantation, conditioned with cyclophosphamide, fludarabine, thiotepa, and total body irradiation (TBI) of 4 Gy. This was complicated by engraftment syndrome for which he is currently being managed. CDI of the patient was corrected by desmopressin administration.

## 1. Introduction

Pituitary involvement has been reported in 0.6% of leukemia cases; however, because pituitary involvement and the presence of diabetes insipidus are not necessarily correlated, the true prevalence of leukemia-associated central diabetes insipidus is unknown [1]. Diabetes insipidus is a condition in which polyuria occurs as the body is unable to maintain the water balance due to either deficiency of ADH (antidiuretic hormone) which is secreted by the pituitary gland or resistance to ADH in the collecting duct of the kidneys. When it occurs in patients with acute leukemia; it is often a poor prognostic indicator [2]. The gene for ADH is located at the cytogenetic location 20p13; however, most reports of patients with acute myeloid leukemia (AML) and diabetes insipidus (DI) describe cytogenetic aberrations of chromosomes 3 and 7 such as inv (3) (q21.3q26.2) and monosomy 7 [2, 3]. Several mechanisms have been proposed to result in pituitary damage [4]. Certain antineoplastic agents have also been implicated in the causation of diabetes insipidus. Here, we report a unique case in which the patient developed central diabetes insipidus with acute myeloid leukemia.

## 2. Case Presentation

This is the case of a 36-year-old healthy male who presented with subacute onset of excessive fatigue, polydipsia, polyuria associated with nocturia, and cold intolerance. On initial workup, his urinalysis was normal with no signs of infection. His complete blood count was significant for leukocytosis (white blood cell: 40,600/*μ*L with 32% neutrophils and 17% blasts in peripheral blood), anemia (hemoglobin: 8.9 g/dl), and a normal platelet count. He was admitted and started on hydroxyurea 500 mg daily, and allopurinol 100 mg daily. A bone marrow aspirate (BMA) and biopsy were performed, and he was discharged on the same day. However, because of increased bleeding from the bone marrow site, he returned to the hospital the day after. The BMA/biopsy results were consistent with chronic myelomonocytic leukemia (CMML-2)/acute myeloid leukemia (AML) with normal karyotype with DNMT3A, CBFB, and PTPN11 mutations. After the course of hydroxyurea, the patient was started on decitabine. He also received continuous intravenous hydration with isotonic fluids and was noted to have a urine output amounting to 12–16 litres per day. The polyuria and polydipsia in conjunction with hypernatremia raised concerns for DI. At baseline, he had a serum osmolality of 297 mOsm/kg, a urine osmolality of 99 mOsm/kg, and the urine sodium was less than 20 mEq/L. The patient underwent a water deprivation test along with administration of one dose of desmopressin acetate (0.05 mg) following which his urine osmolality was found to be 260 mOsm/kg establishing a diagnosis of CDI. Further workup with an MRI (magnetic resonance imaging) of the brain showed focal nodular enhancement at the proximal pituitary infundibulum with a measured value of 0.4 cm with slight effacement of the infundibular recess. The pituitary gland also demonstrated heterogeneous enhancement, and these findings were collectively suggestive of hypophysitis. Additionally, there was a loss of the normal posterior pituitary high-intensity signal on a pre-enhanced T1WI which reflects decreased storage of vasopressin that has a T1-shortening effect in patients with CDI. He was treated with desmopressin and symptomatically improved in the coming months. His urine output during his initial inpatient admission amounted to almost 13-14 liters/day (while receiving maintenance intravenous fluids). With desmopressin, fluid restriction, and diligent follow-up by endocrinologists, his urine output decreased to 5-6 liters/day. He reported improvement in nocturia and polydipsia with the treatments. He also received pituitary radiation for secondary prophylaxis.

## 3. Discussion

AML is a hematologic malignancy contributing to a considerable number of cancer-related deaths. It accounts for approximately 25% of all leukemias in adults in the Western world. The malignancy typically shows 2 peaks in occurrence in early childhood and later adulthood. The etiology for most cases of AML is unclear. A few conditions associated with this neoplasm include genetic disorders (e.g., Down's syndrome, Li-Fraumeni syndrome, and Fanconi Syndrome), exposure to ionizing radiation, chemotherapeutic agents (alkylating agents), certain RNA retroviruses, and secondary AML in the context of a prior history of myelodysplastic syndromes [5].

The combination of AML and DI is unique, and the diagnosis of DI is mostly made either before the diagnosis of AML or at the time of diagnosis of AML [2, 6]. DI with AML is typical of pituitary origin which is supported by the fact that many patients respond to desmopressin [1, 2]. Antidiuretic hormone (ADH) such as vasopressin is produced in the supraoptic nucleus, and it is transported to the posterior pituitary via hypothalamic-neurohypophyseal fibers. DI may result because of infiltration, infarction, infection, hemorrhage, or thrombosis of the pituitary gland [7].

Typically, a bright spot is seen on T1-weighted MRI, due to vasopressin that is stored in the posterior pituitary. It is not necessary for all the patients with central diabetes insipidus (CDI) and AML to have MRI findings; however, patients can have a loss of the neurohypophysis (posterior pituitary) bright spot, which is suggestive of hypothalamic-pituitary dysfunction [2]. Although patients with AML in combination with CDI may have normal MRI findings, it is not necessary that AML patients who have infiltrates in the pituitary always develop CDI [1]. In the case described by Muller et al, the patient with an MRI previously showing absence of the bright spot of the neurohypophysis had reappearance of the bright spot after 2nd dose of induction therapy with FLAG-Ida protocol before allogeneic stem cell transplantation. This encouraged the idea that the infiltrating cells were destroyed due to chemotherapy implicating that infiltration of leukemic cells may be the cause of CDI in AML [8]. A similar finding was seen in our patient's MRI which was suggestive of hypophysitis, pointing towards leukemic infiltration of the posterior pituitary as the cause of CDI.

According to the report of Müller et al, out of 18 cases that were evaluated cytogenetically, 89% had abnormal chromosome 7, 44% had abnormal chromosome 3, and 39% had abnormalities of both chromosome 3 and 7 [8]. In another study conducted by Ladigan et al., cytogenetic analysis of 41/51 patients revealed that monosomy 7 was the most common abnormality, followed by inversion of chromosome 3. Many patients had abnormalities of both chromosomes simultaneously [2].

It is hypothesized that monosomy 7 may lead to abnormal expression of the neutrophil migration gene which causes neutrophil chemotaxis, in turn causing infiltration of the posterior pituitary leading to DI [1, 9]. Further data are needed to determine the role of other molecular phenotypic markers which contribute to leukemic infiltration.

Based on a study, 90% of ADH is bound to platelets, and only 10% is free in the plasma. This is consequential to ADH uptake by platelets from plasma [10]. It is hypothesized that inversion of chromosome 3 causes thrombocytosis due to overexpression of the EVI1 gene eventually leading to interference with ADH levels. When the platelet levels reach the baseline following treatment of the malignancy, the symptoms of DI subside. This stipulates another possible mechanism of DI induced by AML [2, 11, 12]. One case report of CDI with AML had a deletion of chromosome 20q. The latter has a role in the synthesis of ADH, oxytocin, and their carrier protein [7]. Although there has not been enough documentation of this aberration, it is a potential area for upcoming research.

A study by Xu et al. explored the possibility of extramedullary leukemic infiltration mediated by TWIST1 in patients with DNMT3A mutation (which was seen in our patient); however, a definite causal relationship is still unclear [13].

Cytogenetic abnormalities can be detected in approximately 50% to 60% of newly diagnosed AML [14]. Clinical, cytogenetic, and molecular factors have prognostic value. They also help predict treatment resistance which in turn guides therapeutic decisions [15]. A few examples of cytogenetics associated with unfavorable outcomes include monosomies or deletions of part or all of chromosomes 5 or 7 (−5/−7 AML), trisomy 8, and deletion of the long arm of chromosome 20 [14].

Analogously, CDI with AML is an unfavorable association and some reports have documented poor clinical outcomes [2, 6, 16]. Intensive induction chemotherapy and allogeneic stem cell transplantation have been proven to be beneficial in prolonging survival in a few cases [6]. Owing to the polyuria, inaccessibility to water, or inability to drink, patients may develop dehydration and hypotension and patients should be closely monitored for these side effects especially if they are scheduled to receive chemotherapy [1].

From a leukemia standpoint, he progressed to AML from CMML-2 after a cycle of decitabine, with confirmed gingival and presumed central nervous system (CNS) involvement. He is in minimal residual disease (MRD)-negative complete remission after induction with a CNS-active acute lymphoblastic leukemia-2 regimen with high dose cytarabine, mitoxantrone, and intrathecal methotrexate. Most recently, he received double umbilical cord blood transplantation, conditioned with cyclophosphamide, fludarabine, thiotepa, and total body irradiation (TBI) of 4 Gy. This was complicated by engraftment syndrome for which he is currently being managed.

## 4. Conclusion

Endocrine manifestations can be seen in patients with hematologic malignancies. They can be a direct consequence of the malignancy or because of certain chemotherapeutic agents. Their recognition is crucial for prompt management. CDI can be one of the manifestations of AML and carries both therapeutic and prognostic implications. CDI can be managed with desmopressin as seen with our patient. The role of intrathecal chemotherapy and radiation therapy in the pituitary needs to be explored further in adult patients with this presentation.
